# Age and sex related self-reported symptoms in a general population across 30 years: Patterns of reporting and secular trend

**DOI:** 10.1371/journal.pone.0211532

**Published:** 2019-02-04

**Authors:** Annika Bardel, Mari-Ann Wallander, Thorne Wallman, Annika Rosengren, Saga Johansson, Henry Eriksson, Kurt Svärdsudd

**Affiliations:** 1 Uppsala University, Department of Public Health and Caring Sciences, Family Medicine and Preventive Medicine Section, Uppsala University, Uppsala, Sweden; 2 Centre for Clinical Research Sörmland, Uppsala University, Eskilstuna, Sweden; 3 Department of Heart and Lung Diseases, Sahlgren Academy, Gothenburg, Sweden; Texas Technical University Health Sciences Center, UNITED STATES

## Abstract

**Objective:**

To study age and sex specific prevalence of 30 symptoms in random samples from the general population and to analyze possible secular trends across time.

**Study population:**

The study was based on data from eight on-going Swedish cohort studies, with baseline investigations performed between 1973 and 2003. Samples were drawn from the general population of the cities of Gothenburg and Eskilstuna, and of Uppsala County. Overall, 20,160 subjects were sampled, 14,470 (71.8%) responded, of whom 12.000 were unique subjects, and 2548 were part of more than one sample.

**Methods:**

The Complaint score sub-scale of the Gothenburg Quality of Life instrument, listing 30 general symptoms was used. Responders were asked to indicate which symptoms they had experienced during the last three months.

**Results:**

Women reported on average 7.8 symptoms, and men 5.3 (p<0.0001). Women reported higher prevalence than men for 24 of the 30 symptoms. In multivariate analyses four patterns of prevalence across age were identified in both men and women; increasing prevalence, decreasing, stable and biphasic prevalence. The symptoms in the various pattern groups differed somewhat between men and women. However, symptoms related to strain were prominent among symptoms decreasing with age. Moreover, there were secular trends. Across all symptoms reporting prevalence increased over time in men (p<0.001) as well as in women (p<0.0001).

**Conclusions:**

Women reported higher total symptom prevalence than men. Symptoms related to health generally increased with age, while symptoms related to stress decreased markedly. Significant secular trends across time regarding symptom prevalence were found.

## Introduction

According to common knowledge general symptom prevalence should increase by age, presumably because of the ageing process with its wear and tear of body tissues. Since aging populations are becoming a general concern for health care planners it is of interest to know whether this applies to all self-reported symptoms or if it is possible to identify specific patterns of symptom reporting by for instance sex or increasing age.

In an earlier publication [1], based on a subset of the study population used in this report, women 35–64 years of age, we found that the all of the 30 general symptoms listed in the Gothenburg Quality of Life instrument (GQL) did not increase by age. On the contrary, the majority of the symptoms actually decreased by age. There are few other reports on age trends of general symptom prevalence in the general population [2, 3, 4, 5].

In a UK-wide community-based postal survey with a questionnaire covering 25 different symptoms the most commonly reported symptoms were feeling tired/run down, headaches, joint pain, back pain and difficulty sleeping [3]. In that study a population aged 18 to 60 years of age was addressed. The inclusion of persons above 60 was avoided since it was believed that the elderly would present a different symptom profile.

A German study Ladwig et al [4], based on data from a representative health examination survey with 7466 participants in the age range of 25 to 69 years, found a female excess of symptom reporting and utilization of medical services in all age groups. The prevalence of symptom reporting peaked in the age group of 55–59 years followed by a subsequent slight decrease in higher age groups.

A recent Norwegian study by Kjeldsberg et al. [5], using a postal questionnaire on pain related symptoms in a population sample of men and women aged 24 to 86 years of age, revealed that women reported a larger number of symptoms than men irrespective of age. A slight decrease in reporting with age was seen for men but not for women.

Since our previous study [1] did not cover men and did not cover subjects younger than 34 years or older than 64 years, we decided to use data from a number of on-going Swedish cohort studies in the cities of Gothenburg and Eskilstuna, and Uppsala county, which used symptom reporting according to the same protocol as in the previous study. Altogether these studies covered women as well as men and a much wider age range than in the previous study, allowing a more complete age and sex specific symptom prevalence analysis in adult subjects. Furthermore, since data were collected over a time period of thirty years, the dataset allowed estimation of secular trends, i.e., the extent to which symptom-reporting prevalence changes across time, all othrt things being equal [6]. To our knowledge there are no previous publications on secular trends of general symptom reposting in general population samples.

The aim of the present study, based on random samples from the general population, was therefore to investigate the effects of gender, age and year of investigation on symptom-reporting prevalence, after adjustment for the effects of other outcome affecting variables.

## Methods

### Study population

Analyses were based on data from eight on-going Swedish cohort studies, with baseline investigations performed between 1973 and 2003. The study population has previously been described in detail [7, 8]. Briefly, all Swedish residents, whether citizens or not, have a unique personal identification number based on year and date of birth. Systematic or random samples based on predefined specifications concerning age, sex, and area of residence, were drawn from the National Population Register, Table 1.

**Table 1:** Characteristics of the cohorts included in the study population.

Subpopulations	Investigation		Sex	Age range	Sample size	Responders	Response rate,%	Investigation procedure^1)^
	Year	Place						
Men Born in 1913	1973	Gothenburg	Men	60	1009	830	82.3	Q + ME
Men Born in 1913	1980	Gothenburg	Men	67	923	707	76.6	Q + ME
Men Born in 1913	1988	Gothenburg	Men	75	702	463	66.1	Q + ME
Men Born in 1913	1993	Gothenburg	Men	80	447	272	60.9	Q + ME
Men Born in 1923	1973	Gothenburg	Men	50	292	226	77.4	Q + ME
Men Born in 1923	1980	Gothenburg	Men	57	278	188	67.6	Q + ME
Men Born in 1923	1988	Gothenburg	Men	65	265	162	61.1	Q + ME
Men Born in 1923	1993	Gothenburg	Men	70	226	143	63.3	Q + ME
ESKIL	1986	Eskilstuna	Men	30–54	625	459	73.8	PQ
Public Health Cohort	1993	Uppsala	Women	25–99	2999	2249	75,0	PQ
Public Health Cohort	1993	Uppsala	Men	25–94	3001	2156	71.8	PQ
BEDA II	1997	Gothenburg	Women	56–82	994	908	91.3	Q + ME
Uppsala-Örebro Women Study	1995	Uppsala	Women	35–64	4200	2991	71.2	PQ
Men born in 1943	1993	Gothenburg	Men	50	1463	798	54.5	Q + ME
Men born in 1943	2003	Gothenburg	Men	60	749	655	87.4	Q + ME
Women born in 1953	2003	Gothenburg	Women	50	994	668	67.4	Q + ME
Men born in 1953	2003	Gothenburg	Men	50	993	595	59.9	Q + ME
Total					20,160	14,470	71.8	

^1)^ Q = questionnaires; ME = medical examination; PQ = Postal questionnaire

The Men Born in 1913 subpopulation of this study consisted of a systematic third (born on a day divisible by 3 of each month) of the male population aged 60, living in the city of Gothenburg, Sweden, in 1973, Table 1. The Men Born in 1923 subpopulation consisted of a systematic tenth (born on day 3, 15 or 27 of each month) of the male population aged 50, living in Gothenburg in 1973. Survivors in these subpopulations were invited to re-examinations in 1980, 1988 and 1993.

The Men Born in 1943 subpopulation consisted of a random third of 50-year-old men living in Gothenburg in 1993, re-examined in 2003, and the Women and Men Born in 1953 subpopulations consisted of a random third of women and men living in Gothenburg in 2003.

The Eskil subpopulation consisted of a random sample of men aged 30–54 and living in the city of Eskilstuna, Sweden in 1986. The Uppsala Public Health Cohort was based on random samples of men and women 25 years or older from the six municipalities of Uppsala County in 1993.

The Beda II subpopulation was based on a re-examination in 1997 of a random sample drawn in 1979 of women born 1915–1941 and living in Gothenburg. Data from the first examination were not used since no symptom measurements were done. The Uppsala-Örebro Women Study sample was based on random samples of women aged 35–64 from each of the seven counties in the Uppsala-Örebro Health Care Region, Sweden.

All samples were by definition representative of their underlying general populations. By this circumstance it was possible to pool the data, since they were sampled from the same general population. All age groups were not represented on each measurement occasion, and some model-based results might therefore be generated for age groups where no actual measurements were obtained. However, the study aimed at finding trends, and in this respect non-measurements in some time-age groups were of minor importance.

No exclusions were made. The combined samples consisted of 20,160 subjects of whom 3,590 were part of more than one subpopulation (follow-up examinations). Overall, 14,470 (71.8%) observations were obtained, based on 12,000 unique individuals. Of these 10,451 (6,808 women and 3,644 men) participated once, 964 men twice, 254 men three times, and 330 men on four occasions.

### Measurements

The data used in this report was obtained in some of the studies by questionnaire in conjunction with medical examinations performed, in others by postal questionnaire. All questionnaires, whether distributed at home or at screening sites, were filled in solitude. No interviews were performed regarding the variables used in this report, which means that responses from questionnaire filled in at home or at screening sites should be equivalent. For the variables used here the same questionnaires were used in all studies. Educational level was classified on a four-point scale ranging from ‘compulsory education only’ (= 1), to ‘college or university education’ (= 4). Marital status was classified as married/cohabiting or not (the latter including response alternatives never married, divorced, and widowed). Height was measured to the nearest centimeter, using a measuring stick mounted on a wall and weight on a lever balance to the nearest tenth of a kilogram. Based on these two variables body mass index (BMI) (weight (kg)/height (m)^2^) was computed.

Symptom reporting was assessed based on the Complaint Score scale of The Gothenburg Quality of Life instrument (GQL). The scale was evaluated in 1990 regarding reproducibility, which was excellent, and relation to other more hard-core measures of disease, which was acceptable [9]. Subjects were asked ‘Have you been troubled by any of the following symptoms during the past three months?’ followed by a list of 30 general symptoms with response alternatives ‘yes’ (= 1) or ‘no’ (= 0) for each symptom. The Complaint score was obtained as the sum of yes-answers across the 30 symptoms. Smoking habits were classified as ‘current smoker’ or ‘non-smoker’ (including never smoked and ex-smoker). In addition, in some of the cohorts a five-point smoking variable was available, where smoking habits were classified as ‘never smoked’ (= 1), ‘ex-smoker’ (= 2), ‘currently smoking 1–14 grams of tobacco per day’ (= 3), ‘smoking 15–24 grams per day’ (= 4), or ‘smoking 25 grams or more per day’, with one cigarette equaling 1 gram, one cheroot 2 grams, one cigar 5 grams, and pipe tobacco 50 grams, divided by the number of days the pack lasted [10].

### Ethical considerations

All participants gave informed consent, verbal in the earlier studies, and written later on, as required first by the Research Ethics Committees at Gothenburg and Uppsala Universities and later by the National Research Ethics Board. The Committees and the Board approved the study on several occasions during the data collection process.

### Statistical analyses

Data were analyzed with the SAS software, version 9.3 [11]. Data concerning age, sex and examination year were complete, except for one individual where age was missing. Not all variables were measured in all subpopulations. The number of available observations for each variable is shown in Table 2. The overall proportion of missing data in subpopulations where the variables were measured was less than 2%. Missing data were not replaced. Simple differences between the sexes were tested with Student’s t-test for continuous variables and the chi-square test for discrete variables.

**Table 2:** Characteristics of the study population.

		N	Women			Men			
			n	Mean or %	SD	n	Mean or %	SD	p
Number of observations		14,470	6,816	47.1		7,654	52.9		
Mean follow-up time, years				8.6	2.6		11.4	5.6	
Person-years during follow up			27,034			73,217			
Age, years		14,469	6,816	52.3	12.6	7,653	56.5	13.0	<0.0001
Education		14,120	6,722			7,398			<0.0001
	Compulsory school only, %		2,223	33.1		2,773	37.5		
	Vocational school, %		1,608	23.9		1,905	25.8		
	Upper secondary school, %		1,286	19.1		1,166	15.8		
	University/college, %		1,605	23.9		1,554	21.0		
Occupational status		14,187	6,575			7,612			<0.0001
	Unemployed, %		271	4.1		286	3.8		
	Sick-leave/disability pension, %		713	10.8		624	8.2		
	Old age retirement, %		1,147	17.4		2,191	28.8		
Married/cohabiting. %		14,338	4,990	74.0		5,927	78.0		<0.0001
Smoking habits		14,330	6,735			7,595			0.001
	Never smoked or ex-smoker, %		5,002	74.3		5,455	71.8		
	Current smoker, %		1,733	25.7		2,140	28.2		
Leisure time physical activity		13,787	6,678			7,109			<0.0001
	Sedentary, %		1,076	16.1		1,181	16.6		
	Moderately active, %		4,663	69.8		4,511	63.4		
	Active, %		890	13.3		1,304	18.3		
	Vigorously active, %		49	0.7		113	1.6		
Body mass index			6,816	24.7	3.47	7,654	25.6	2.82	<0.0001
Complaint score (range 0–30)		11,365	3,777	7.8	5.4	7,588	5.3	4.7	<0.0001

Sex differences were tested with Student’s t-test for continuous variables and the chi-square test for discrete variables.

The final analysis model was based on multiple nominal logistic regression analyses with presence of reported symptom (yes or no) as outcome (dependent) variable and age-group (decades), examination year, educational level, BMI, marital status and smoking habits as exposure (independent) variables, with backward elimination of non-significant covariates. To facilitate the analyses and descriptions the same covariates were used for all symptoms. Possible non-linearity of the effects of age and year of investigation on the 30 symptoms was tested by inclusion of the variables age and year of investigation, respectively, raised to the power of 2 (second degree polynomial function) and to the power of 3 (third degree polynomial function). Moreover, potential effects of interaction between age and year of investigation were tested, but none was found.

Two measures of degree of explanation are provided by the SAS logistic procedure, one based on concordance between observed and model computed results, the other based on the concordance index [12]. Both exceeded 70%. The model fit between crude age and symptom prevalence and those computed in the final analytical model was assessed by scrutiny and found to be close.

Possible secular trends regarding symptom reporting were tested with multiple linear regression analysis with complaint score as dependent variable and year of examination as independent variable, adjusted for the influence of the variation of distribution of age, sex, education, marital status, body mass index and smoking habits across the study period.

The study design was a mixed cross-sectional and longitudinal one, since the subpopulations ‘Study of Men Born in 1913’ and ‘Study of Men Born in 1923’ were re-examined three times with 5–7 years intervals, and the subpopulation ‘Study of Men Born in 1943’ was re-examined one time after ten years. To check the influence of the mixed design as compared to a cross-sectional one a sensitivity analysis was performed comparing the results of only cross-sectional data with the actual mixed design. The sensitivity analysis showed the same results for both types of design, indicating that the inclusion of repeated measures with the time intervals used had no effect on the results over and above the cross-sectional design.

The attrition rate was a moderate 28%. To test the potential effect of attrition on the secular trend slope, results were re-analyzed based on the assumption that non-participants in the early and the late parts of the study period reported considerably lower or higher levels (+5 or -5 standard error units) of symptoms than participants, but no significant effect of attrition rate on secular trend slope was found. All tests were two-tailed. Significance levels were set at p< 0.05.

## Results

### Characteristics of the study population

Mean age for men and women combined was 54.5 years. Women were significantly younger than men and had higher level of education, were less often married/cohabiting, less often current smokers, less physically active, and had lower BMI than men, Table 2. On average, women reported higher symptom prevalence than men (mean complaint score in women 7.8 and in men 5.3).

### Symptom prevalence

**Fig 1:**
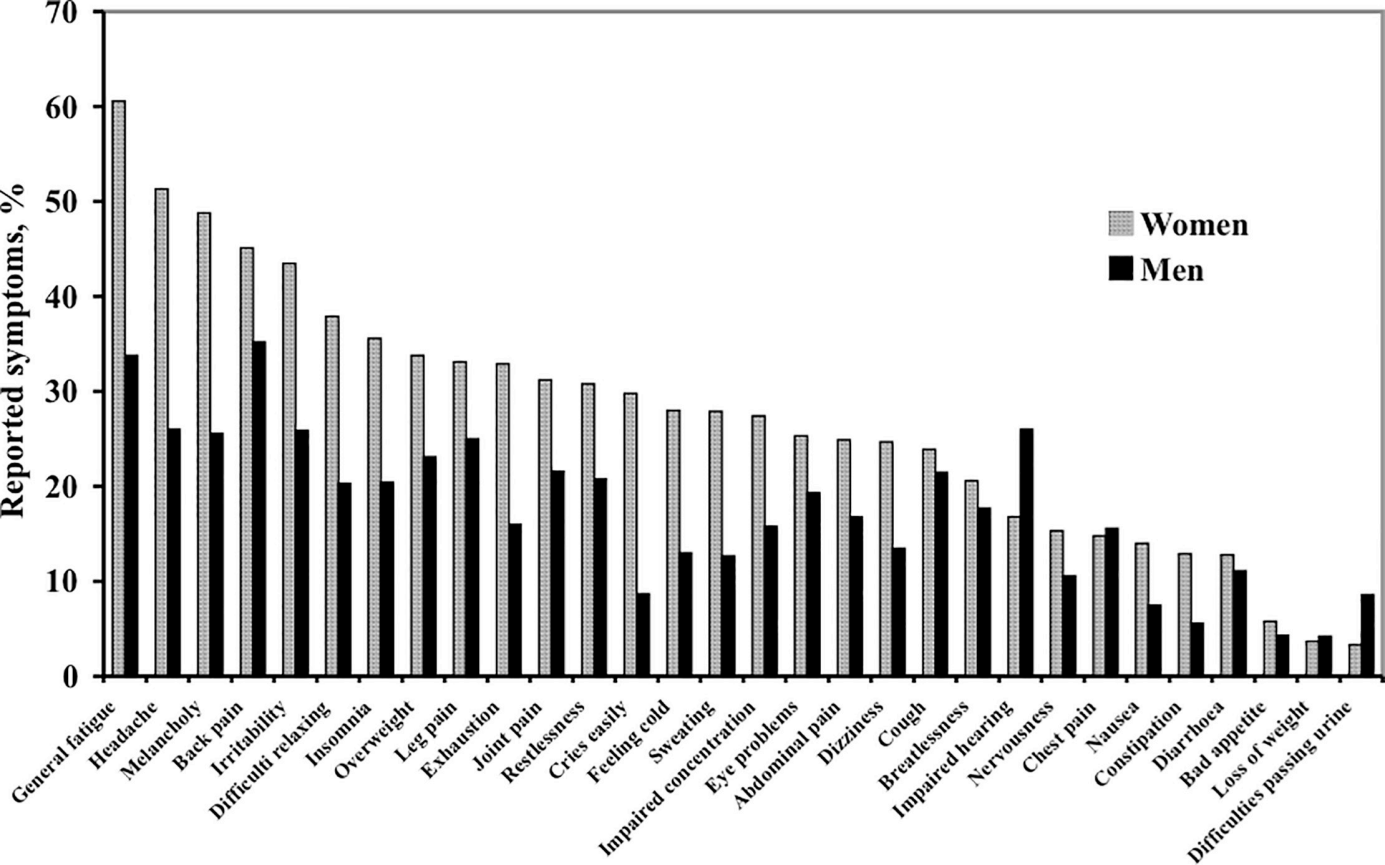
Symptom prevalence among men and women, adjusted for age, examination year, education, BMI, marital status and smoking habits and ranked according to the level in women. All differences were significantly different (p<0.0001), except for ‘poor appetite’ (p<0.05) and ‘chest pain’, ‘cough’, ‘diarrhea’ and ‘loss of weight’ (no significant difference).

The symptom prevalence by sex adjusted for the influence of age, displayed in Fig 1, showed that women reported higher prevalence than men for 24 of the 30 symptoms, men reported higher prevalence for two symptoms (‘impaired hearing’ and ‘difficulty passing urine’), and for four symptoms (‘chest pain’, ‘cough’, ‘diarrhoea’, and ‘loss of weight’) no significant differences were found.

The age trends for the 30 general symptoms among women after adjusting for examination year, education, BMI, marital status and smoking are shown in Table 3. The prevalence of eight symptoms increased significantly by age. Most of these were well-known health problems coming with advancing age. The most prevalent were ‘insomnia’, ‘joint pain’, ‘leg pain’ and ‘eye problems’, including impaired vision, that were reported by 28% or more among those aged 50 and older.

**Table 3:** Multivariate analyses of increasing, stable, biphasic and decreasing symptom prevalence in women.

		Age groups, years							
		25–29	30–39	40–49	50–59	60–69	70–79	80+	p*^)^
N		221	862	1645	2168	1279	480	161	
**Increasing prevalence, %**									
	Insomnia	18.1	29.1	34.3	43.9	43.3	49.4	45.0	<0.0001
	Joint pain	10.4	18.7	28.8	40.1	32.2	29.8	38.3	<0.0001
	Leg pain	12.7	21.0	28.4	39.5	36.6	36.3	53.2	<0.0001
	Eye problems	14.9	16.1	29.8	28.1	33.7	50.0	91.6	<0.0001
	Breathlessness	8.6	13.5	17.7	18.1	21.9	24.5	35.0	<0.0001
	Dizziness	24.4	23.2	23.4	17.9	17.2	25.5	40.7	<0.0001
	Chest pain	5.4	12.3	16.5	16.2	15.0	18.5	17.6	0.04
	Impaired hearing	6.8	7.9	10.9	15.6	25.4	36.3	92.1	<0.0001
**Stable prevalence, %**									
	Nervousness	8.6	13.5	14.3	13.4	12.0	11.9	12.6	0.64
	Constipation	10.0	11.9	11.8	12.4	11.9	11.0	19.0	0.63
	Loss of weight	3.2	4.2	3.7	2.9	2.6	6.1	10.7	0.13
	Difficulty passing urine	0.1	0.2	0.2	0.3	0.2	0.4	0.2	0.14
**Biphasic prevalence, %**									
	Sweating	9.0	13.1	30.5	47.8	20.4	13.5	7.2	<0.0001
	Overweight	21.3	24.2	25.8	34.6	25.4	14.5	7.4	<0.0005
	Impaired concentration	26.2	27.6	36.8	25.7	16.4	10.5	11.9	<0.0001
**Decreasing prevalence %**									
	Difficulty relaxing	34.8	36.5	45.7	43.1	32.2	29.1	18.3	<0.0001
	Melancholy	52.0	55.2	59.2	41.2	29.4	30.5	24.8	<0.0001
	Back pain	47.1	42.0	39.3	39.6	32.8	25.0	21.1	<0.0001
	General fatigue	79.2	81.4	58.3	37.4	17.7	15.9	22.1	<0.0001
	Exhaustion	40.3	45.6	51.3	35.6	13.9	9.7	9.4	<0.0001
	Cough	26.2	27.5	26.4	26.0	25.4	19.7	14.4	0.04
	Restlessness	39.4	28.3	34.4	24.6	18.0	14.8	5.6	<0.0001
	Irritability	64.7	60.6	44.3	23.7	13.7	8.6	8.2	<0.0001
	Headache	76.9	61.4	43.5	22.8	11.4	8.2	6.4	<0.0001
	Feeling cold	31.2	33.6	33.2	19.7	13.3	15.3	21.4	<0.0001
	Abdominal pain	32.6	29.9	24.2	19.4	15.6	11.5	11.1	<0.0001
	Cries easily	36.2	31.8	25.5	18.6	12.7	11.1	13.7	<0.0001
	Diarrhea	11.3	16.3	14.9	13.2	11.8	9.6	7.6	<0.01
	Nausea	22.6	18.8	14.4	10.7	7.8	5.8	6.0	<0.0001
	Poor appetite	12.7	6.8	5.4	3.7	3.2	4.6	8.9	<0.01

*^)^ p values refer to trends across age after adjustment for the influence of the covariates examination year, education, BMI, marital status and smoking in logistic regression models. The percentages were computed based on odds ratios.

The prevalence of four symptoms did not vary significantly across age. The most common were ‘nervousness’ and ‘constipation’, affecting on average 12%-13% of the women. The prevalence of three symptoms, ‘sweating’, ‘overweight’, and ‘impaired concentration’, had a biphasic course with an increase until age 50–59 and from then on a decrease.

The prevalence of fifteen symptoms decreased by age. Most of these were potentially related to strain and stress. The most common symptoms in this group were ‘difficulty relaxing’ and ‘melancholy’, both of which displayed a peak at age 40–49 and then decreased, while ‘back pain’, and ‘general fatigue’, reported by 35%-79% of women under 50 years of age generally decreased with age.

The corresponding data for men, displayed in Table 4, showed the same pattern with four prevalence course groups. The symptoms in the group with increasing prevalence across age were largely the same as in women. However, the group of symptoms with stable prevalence, ‘chest pain’ and ‘feeling cold’ were different from stable prevalence symptoms in women. Of the symptoms with biphasic prevalence course two symptoms, ‘sweating’ and ‘overweight’ were the same as in women, but the remaining three, ‘exhaustion’, ‘nervousness’, and ‘loss of weight’ were different.

**Table 4:** Multivariate analyses of increasing, stable, biphasic and decreasing symptom prevalence in men.

		Age groups, years							
		25–29	30–39	40–49	50–59	60–69	70–79	80+	p*^)^
N		243	612	680	2263	2636	856	363	
**Increasing prevalence, %**									
	Leg pain	11.9	20.0	24.7	21.0	24.2	24.5	41.4	<0.0001
	Joint pain	11.9	17.8	20.4	20.1	25.2	19.7	20.8	<0.005
	Impaired hearing	9.9	11.2	15.7	18.4	31.7	52.1	80.5	<0.0001
	Eye problems	10.7	15.8	25.4	17.1	19.5	28.8	53.5	<0.0001
	Breathlessness	9.1	9.5	13.2	12.6	15.6	20.7	33.1	<0.0001
	Dizziness	13.6	16.1	14.4	10.1	11.7	13.4	32.4	<0.0001
	Cries easily	6.2	6.3	6.5	7.7	8.5	8.9	11.1	<0.01
	Constipation	2.1	3.9	4.7	5.0	6.0	9.1	17.9	<0.0001
	Difficulty passing urine	0.01	1.2	3.2	5.8	12.2	12.8	17.7	<0.0001
**Stable prevalence, %**									
	Chest pain	13.6	19.1	16.0	13.6	12.5	13.0	15.7	0.09
	Feeling cold	15.6	13.9	10.9	10.2	9.7	14.1	18.7	0.17
**Biphasic prevalence, %**									
	Exhaustion	24.7	40.0	36.0	18.7	9.9	4.6	6.2	<0.0001
	Overweight	9.1	22.5	25.7	17.6	16.2	12.4	7.4	<0.0001
	Sweating	10.7	10.8	9.5	13.6	10.5	9.5	9.0	<0.05
	Nervousness	8.6	10.8	11.2	11.6	9.0	6.6	7.8	<0.0005
	Loss of weight	5.3	4.3	2.6	2.6	4.4	5.4	10.4	<0.001
**Decreasing prevalence %**									
	Back pain	35.0	44.0	46.3	33.7	32.0	21.7	23.3	<0.0001
	Melancholy	36.2	40.0	41.8	23.9	19.5	15.2	19.9	<0.0001
	Difficulty relaxing	27.6	36.1	31.2	22.6	15.3	9.8	11.1	<0.0001
	Irritability	39.9	52.0	40.9	22.6	16.5	12.1	10.8	<0.0001
	Insomnia	21.8	25.9	28.5	22.2	21.9	16.2	19.8	<0.005
	General fatigue	65.0	70.4	39.0	18.1	12.7	9.5	13.1	<0.0001
	Restlessness	40.7	36.6	28.4	17.4	12.6	7.4	10.9	<0.001
	Impaired concentration	25.5	29.9	30.6	17.0	12.1	7.6	11.8	<0.0001
	Headache	58.0	50.7	37.2	16.9	9.2	5.8	4.8	<0.001
	Cough	31.7	31.8	20.3	16.0	14.5	12.5	14.3	<0.0001
	Abdominal pain	26.7	27.5	17.8	15.3	10.5	8.4	9.5	<0.0001
	Diarrhea	18.5	16.8	15.1	14.0	8.9	5.1	5.5	<0.0001
	Nausea	14.8	14.6	11.0	6.2	4.5	3.2	4.7	<0.0001
	Poor appetite	8.2	6.3	4.8	3.5	3.3	2.8	4.7	<0.005

*^)^ p values refer to trends across age after adjustment for the influence of the covariates examination year, education, BMI, marital status and smoking in logistic regression models. The percentages were computed based on odds ratios.

As among women, almost half of the symptoms had a decreasing prevalence course also among men. Most of these were potentially related to strain and stress. The most common symptoms were ‘back pain’, ‘melancholy’, ‘difficulty relaxing’, ‘irritability’, and ‘general fatigue’, affecting a substantial proportion of men under the age of 50 years and from then on decreased.

**Fig 2:**
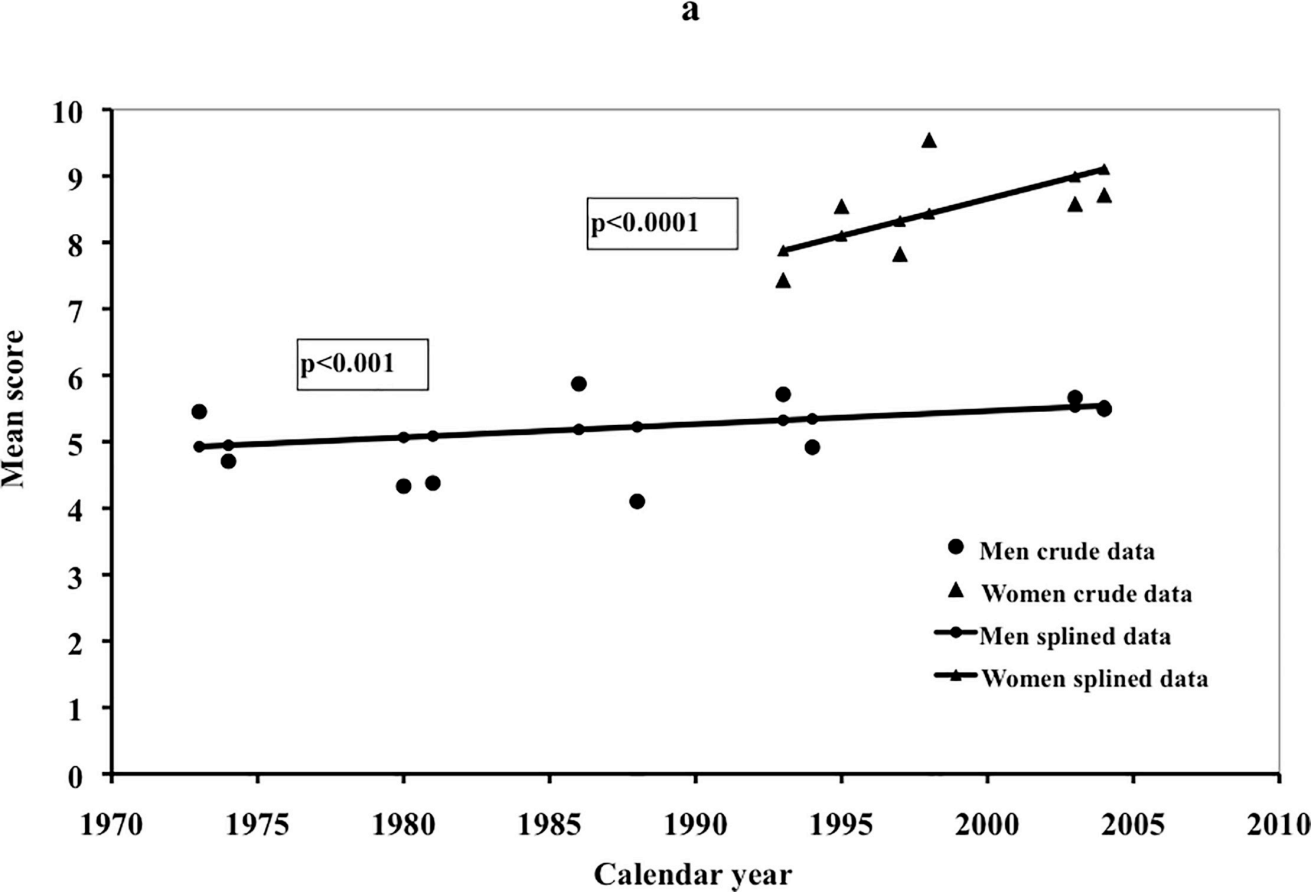
Crude and smoothed trends of symptom reporting across time among men and women for all Complaint Score symptoms combined, controlling for age, education, BMI, marital status and smoking habits.

Possible secular trends, i.e., changing tendency to report symptoms across time in men and women after taking the influence of age into account, is shown in Fig 2. Overall reporting of symptoms increased among men as well as among women (Fig 2).

## Discussion

Women reported higher total symptom prevalence than men. There were gender differences seen in prevalence patterns across age among 10 of the 30 reported symptoms. Four symptom prevalence patterns were found, increasing, stable, biphasic, and decreasing. Generally there was a secular trend towards a tendency of increased reporting across time after taking the effects of age into account. However, for different symptom groups the secular trends were at variance.

The main strength of the study concerns sample size. Data were based on eight on-going population studies, covering more than 14 000 observations scattered over a 30-year period. Pooling of the data was justified by the fact that all samples were randomly drawn from the general population in three geographical areas. At the time of sampling the Swedish population was rather homogenous with a small fraction of immigrants. The sample size could thus be made larger than in any previous study investigating effect of age, sex and year of investigation on symptom reporting, and observation time spanned up to three decades in men and one decade in women. The determinant variables were measured with the same instruments in all cohorts.

The limitation of the study was that all age groups were not represented on each measurement occasion, and some model-based results were thus generated for age groups where no actual measurements were obtained. However, this is a problem common in multiple analysis modeling, and not unique to this study. On the other hand, in the analyses of symptom reporting by sex, the variables age and year of investigation were taken into account, which lessens the negative effects of model-based results.

We used a mixed cross-section and longitudinal design. A sensitivity analysis indicated results similar to those from a cross-sectional design. There was no significant effect of attrition on the secular trend slope. We have therefore no reason to believe that the results are afflicted by selection or other bias to such an extent that the conclusions would be affected.

Few other population-based studies have looked at a similar range of non-specific symptoms in relation to age and sex [1, 2, 3, 4, 5]. The present study is so far the largest and has the widest age span. To our knowledge there are no published population based studies available where secular trends in the reporting of non-specific symptoms have been studied.

Women reported higher total symptom prevalence than men. This is common medical knowledge and in accordance with findings from other studies [3, 4, 5]. Despite the fact that women reported higher symptom prevalence, the pattern of reported symptoms was similar in women and men, with the most frequently reported symptoms being ‘general fatigue’, ‘headache’ and ‘back pain’ after adjustment for age. The findings are in agreement with findings from other studies [1, 3, 13].

Four symptom patterns were seen in the study, increasing, decreasing, stable and bi-phasic. As expected, the symptoms that increased with age were those known to be associated with health problems in older age like deteriorated vision and hearing, joint and leg pain and in women also insomnia.

That insomnia is more common in women than in men has been shown previously [14, 15, 16] and many attempts have been made to explain the gender difference in sleeping patterns by for instance biological factors and gender differences in reporting. However, the explanation might be multi-factorial and seems to vary across age groups. Further research is needed to fully understand the differences in sleeping pattern between women and men.

Additionally, men showed an increasing reporting pattern for the symptom ‘cries easily’ moving from a very low prevalence in young age to become similar to that in women in the oldest age group while women showed a stable pattern. Another symptom with very different patterns between men and women was ‘difficulty in passing urine’ which showed an increasing pattern in men while its prevalence remained stable in women. It is difficult to discuss these findings in relation to other studies since focus generally has been put on gender differences in symptom counts rather than on individual symptom patterns in relation to sex and age.

A large number of symptoms showed a decreasing reporting prevalence with age in women as well as men, in agreement with earlier findings (4). The symptoms in this group were mainly associated with stress and strain like for instance ‘difficulty relaxing’, ‘melancholy’, ‘irritability’, ‘headache’ and ‘back pain’. The high reporting prevalence in young age might be due to a high stress level in daily life in young people who perhaps have small children and are to be established on the labor market. In older ages these stress levels might gradually decrease which might result in decreasing prevalence rates of these symptoms.

The increased knowledge about different symptom reporting patterns over age could lead to a modified and more positive view on aging. The total symptom burden does not seem to increase with increasing age but quite a lot of symptoms actually decrease with aging. Sweden like most western countries are facing an aging population with an anticipated increased strain on the health care system. Learning more about what kind of symptoms that actually are becoming worse with aging might help in future planning of resources.

The secular trends found are novel. As far as we know no previous published study has reported similar findings. The overall tendency was a moderate but significant increase of symptom reporting prevalence across time in men (p<0.001), and a more steep increase in women (p<0.0001).

The reasons for the secular trends remain to be elaborated on. However, when the Complaint score instrument was constructed, the underlying goal was to create an easy-to-use quality of life instrument (personal communication Susanne Ander Peciva and Bodil Tibblin). Complaint score does not measure presence or absence of disease. Most people may have many of the symptoms included in Complaint score during a three-month period without regarding themselves as being ill. It rather measures the tendency to report symptoms. This tendency is supposed to increase in periods of stress, or unease, and to decrease in periods of less pressure. In this sense it might be a measure of quality of life. It may therefore be that modern lifestyle with its increasing pace in working as well as in private life, causes frustration and thereby more symptom reporting across time, i.e., a positive secular trend.

## Conclusions

Women reported higher total symptom prevalence than men. There were gender differences for 10 of the 30 reported symptoms, while 20 of the examined symptoms had similar prevalence patterns across age. Four symptom patterns were found, increasing, decreasing, stable, and biphasic. About half of the symptoms decreased by age. A secular trend was found showing an increase in symptom reporting from 1985 and onwards.

## Supporting information

S1 File The file named [dataset.sas7bdat] contains the data set on which this study is based. (SAS7BDAT)
